# 2021 ACC/AHA/SVM/ACP Advanced Training Statement on Vascular Medicine (Revision of the 2004 ACC/ACP/SCAI/SVMB/SVS Clinical Competence Statement on Vascular Medicine and Catheter-Based Peripheral Vascular Interventions)

**DOI:** 10.1161/HCV.0000000000000079

**Published:** 2021-01-15

**Authors:** Mark A. Creager, Naomi M. Hamburg, Keith D. Calligaro, Ana I. Casanegra, Rosario Freeman, Phyllis A. Gordon, Heather L. Gornik, Esther S.H. Kim, Nicholas J. Leeper, Geno J. Merli, Khusrow Niazi, Jeffrey W. Olin, Rene Quiroz, Elona Rrapo Kaso, Suman Wasan, Andrew R. Waxler, Christopher J. White, Khendi White Solaru, Marlene S. Williams

**Affiliations:** * Society for Vascular Surgery representative.; † Society for Vascular Medicine representative.; ‡ Society for Vascular Nursing representative.; § American Heart Association representative.; ‖‖ American College of Physicians representative.; ¶ Society for Cardiovascular Angiography and Interventions representative.; # Association of Black Cardiologists representative.

**Keywords:** AHA Scientific Statements, aortic diseases, cerebrovascular disease, clinical competence, fellowship training, lymphedema, peripheral artery disease, Raynaud phenomenon, vascular medicine, venous insufficiency, venous thromboembolism

## Abstract

Supplemental Digital Content is available in the text.

## Table of Contents

Preamble 241
1. Introduction 242
  1.1. Document Development Process 242
    1.1.1. Writing Committee Organization 242
    1.1.2. Document Development and Approval 242
  1.2. Background and Scope 242
    1.2.1. Evolution of Vascular Medicine 243
    1.2.2. Levels of Training 243
2. General Standards 243
  2.1. Faculty 243
  2.2. Facilities 244
  2.3. Equipment 244
  2.4. Additional Resources 244
3. Training Components 244
  3.1. Didactic Program 244
  3.2. Clinical Experience 245
  3.3. Hands-On Procedural Experience 245
  3.4. Diagnosis and Management of Emergencies and Complications 246
  3.5. Diagnosis and Management of Less Common Clinical Conditions and Syndromes 246
  3.6. Research and Scholarly Activity 246
4. Training Requirements 246
  4.1. Development and Evaluation of Core Competencies 246
    Table 1. Competency Components and Curricular Milestones for Level III Training in Vascular Medicine 247
    Table 2. Common Professional Behavior Competencies Relevant to All Clinical Cardiovascular Disease Specialists 251
  4.2. Vascular Medicine 250
    4.2.1. Peripheral Artery Disease 250
    4.2.2. Renal and Mesenteric Artery Disease 250
    4.2.3. Cerebrovascular Disease 252
    4.2.4. Aortic Diseases 252
    4.2.5. Vasculitis, Vasospastic, and Temperature-Related Disorders 252
    4.2.6. Acute and Chronic Venous Diseases 252
      4.2.6.1. Acute Venous Disease 252
      4.2.6.2. Chronic Venous Disease 253
    4.2.7. Lymphatic Diseases and Lipedema 253
    4.2.8. Noninvasive Diagnostic Tests 253
5. Leadership and Administrative Competencies 254
6. Evaluation of Proficiency 254
References 256
Appendix 1. Author Relationships With Industry and Other Entities (Relevant) 256
Appendix 2. Peer Reviewer Information 258
Appendix 3. Abbreviations 259

## Preamble

Since publication of its first Core Cardiovascular Training Statement (COCATS) in 1995,^1^ the American College of Cardiology (ACC) has defined the knowledge, experiences, skills, and behaviors expected of clinical cardiologists. Subsequent revisions have moved toward competency-based training based on the 6-domain competency structure promulgated by the Accreditation Council for Graduate Medical Education (ACGME) and the American Board of Medical Specialties and endorsed by the American Board of Internal Medicine (ABIM). The ACC has taken a similar approach to describe the aligned general cardiology lifelong learning competencies that practicing cardiologists are expected to maintain. Many hospital systems now use the 6-domain structure as part of medical staff privileging, peer review, and professional competence assessments.

Whereas COCATS and the associated Lifelong Learning Competencies for General Cardiologists^2^ focus on general clinical cardiology, ACC Advanced Training Statements and associated Lifelong Learning Statements define selected competencies beyond those expected of all cardiologists and that typically require training beyond a standard 3-year cardiovascular disease fellowship. This includes, but is not limited to, those disciplines for which there is an ABIM subspecialty certification. The Advanced Training Statements describe key experiences and outcomes necessary to acquire skills in a defined subspecialty area of cardiology in a structured training program. These are supplemented by Lifelong Learning Statements that address the commitment to sustaining and enriching competency over the span of a career.

The ACC Competency Management Committee oversees the development and periodic revision of the cardiovascular training and competency statements. A key feature of competency-based training and performance is an outcome-based evaluation system. Although specific areas of training may require a minimum number of procedures or duration of training to ensure adequate exposure to the range of clinical disorders, the objective assessment of proficiency and outcomes demonstrates the achievement of competency. Evaluation tools include examinations, direct observation, procedure logbooks, simulation, conference presentations, and multisource (360°) evaluations. For practicing physicians, these tools also include professional society registry or hospital quality data, peer review processes, and patient satisfaction surveys. A second feature of competency-based training is recognition that learners gain competency at different rates. For multiyear training programs, assessment of representative curricular milestones during training can identify learners or areas that require additional focused attention.

The recommendations in ACC Cardiovascular Training and Lifelong Learning Statements are based on available evidence and, where evidence is lacking, reflect consensus expert opinion. The writing committees are broad-based, and typically include early-, mid-, and later-career specialists; general cardiology and subspecialty training directors; practicing cardiologists; people working in institutions of various sizes and in diverse practice settings across the United States; and nonphysician members of the cardiovascular care team. All documents undergo a rigorous process of peer review and public comment. Recommendations are intended to guide the assessment of competence of cardiovascular care providers beginning independent practice as well as those undergoing periodic review to ensure that competence is maintained.

This Advanced Training Statement addresses the core competencies required of vascular medicine specialists and complements the training in vascular medicine required of all physician trainees during the standard 3-year general cardiovascular fellowship. Furthermore, this statement identifies selected competencies of vascular medicine specialists that go beyond core expectations that may be achieved by some advanced trainees either during formal fellowship training or through subsequent training experiences. This document provides examples of appropriate measures for assessing competence in the context of training.

The work of the writing committee was supported exclusively by the ACC without commercial support. Writing committee members volunteered their time to this effort. Conference calls of the writing committee were confidential and attended only by committee members. To avoid actual, potential, or perceived conflict of interest resulting from relationships with industry (RWI) or other entities held by writing committee members or peer reviewers of the document, individuals were required to disclose all current healthcare-related relationships, including those existing 12 months before initiation of the writing effort. The ACC Competency Management Committee reviewed these disclosures to identify products (currently marketed or under development) pertinent to the document topic. Based on this information, the writing committee was selected to ensure that the chair and a majority of members had no relevant RWI. RWI was reviewed at the start of all meetings and conference calls and updated as changes occurred. Relevant RWI for authors is disclosed in Appendix 1. To ensure transparency, comprehensive RWI for authors, including RWI not pertinent to this document, is posted in Supplemental Appendix I. Employment information and affiliations of the peer reviewers are shown in Appendix 2. There are no RWI restrictions for participation in peer review, in the interest of encouraging comments from a variety of constituencies to ensure that a broad range of viewpoints inform final document content. Reviewers are required, however, to disclose all healthcare-related RWI and other entities, and their disclosure information is posted in Supplemental Appendix II. Disclosure information for the ACC Competency Management Committee is available online as well as the ACC disclosure policy for document development.

*James A. Arrighi, MD, FACC*

*Chair, ACC Competency Management Committee*

*Lisa A. Mendes, MD, FACC*

*Co-Chair, ACC Competency Management Committee*

## 1. Introduction

### 1.1. Document Development Process

#### 1.1.1. Writing Committee Organization

The writing committee consisted of a broad range of members representing the ACC, American Heart Association (AHA), American College of Physicians (ACP), Society for Vascular Medicine (SVM), Association of Black Cardiologists, Society for Cardiovascular Angiography and Interventions, Society for Vascular Nursing, and Society for Vascular Surgery. Each performs at least 1 of the following roles: 1) vascular medicine specialists who work in academic and/or community-based practice settings, and who represent early to late career professionals; 2) training program directors of vascular medicine and of cardiovascular medicine; and 3) other specialists representing general cardiology, interventional cardiology, vascular surgery, vascular nursing, vascular research, and advanced practice nurses. The writing committee also included physicians experienced in defining and applying training standards according to the 6 general competency domains promulgated by the ACGME and the American Board of Medical Specialties and endorsed by the ABIM. This writing committee met the College’s disclosure requirements for RWI as described in the Preamble.

#### 1.1.2. Document Development and Approval

The writing committee convened by conference call and email to finalize the document outline, develop the initial draft, revise the draft based on committee feedback, and ultimately approve the document for external peer review.

The document was reviewed by 18 official representatives from the ACC, AHA, ACP, SVM, Association of Black Cardiologists, Society for Cardiovascular Angiography and Interventions, Society for Vascular Nursing, and the Society for Vascular Surgery, as well as by 25 additional content reviewers (see Appendix 2). The document was simultaneously posted for public comment from February 26, 2020, to March 11, 2020. A total of 409 comments were submitted on the document. All comments were reviewed and addressed by the writing committee. A member of the ACC Competency Management Committee served as lead reviewer to ensure a fair and balanced peer review resolution process. Both the writing committee and the ACC Competency Management Committee approved the final document to be sent for organizational approval. The ACC, AHA, ACP, and SVM approved the document for publication with endorsement from the Association of Black Cardiologists, Society for Cardiovascular Angiography and Interventions, Society for Vascular Nursing, and Society for Vascular Surgery. This document is considered current until the ACC Competency Management Committee revises or withdraws it from publication.

### 1.2. Background and Scope

The original 1995 American College of Cardiology recommendations for training in adult cardiology evolved from a Core Cardiology Training Symposium.^1^ After several iterations, COCATS 4 focuses on trainee outcomes that require delineation of specific components of competency within the subspecialty, definition of the tools necessary to assess training, and establishment of milestones documenting the trainee’s progression toward independent competency.^3^ Ultimately, the goal is for the trainee to develop the professional skill set to be able to evaluate, diagnose, and treat patients with acute and chronic cardiovascular disturbances.

COCATS 4 includes individual task force reports that address subspecialty areas in cardiology, each of which is an important component in training a fellow in cardiovascular disease. The Task Force 9 report of that document addressed training in vascular medicine and updated previous standards for general cardiovascular training for fellows enrolled in cardiovascular fellowship programs.^4^ It addressed faculty, facilities, equipment, and ancillary support. It also addressed training components, including didactic, clinical, and hands-on experience, and the number of procedures and duration of training to acquire core Level I and II competencies in vascular medicine. Importantly, the COCATS 4 Task Force 9 report did not provide detailed guidelines for Level III training in vascular medicine, but described it in broad terms to provide context for trainees and clarify that these advanced competencies are not covered during the cardiovascular fellowship. Given the burden of vascular diseases, it is critical that trainees in cardiovascular medicine receive appropriate training in vascular medicine as described in COCATS 4 and have the opportunity to pursue advanced training in this field.

This Advanced Training Statement focuses on training requirements for physicians seeking additional training in vascular medicine. It describes the pathway in which these advanced skills are acquired following a cardiovascular fellowship, but it can also be applied to trainees who pursue advanced training in vascular medicine without first specializing in cardiovascular medicine. Training requirements for physicians seeking competency in endovascular interventions will be addressed in a future advanced training statement.

#### 1.2.1. Evolution of Vascular Medicine

The responsibilities of vascular medicine physicians range from preserving vascular health and treating vascular diseases to advancing vascular science. Although cardiovascular physicians frequently encounter and care for patients with a wide range of arterial and venous disorders, the management of vascular disease often necessitates highly trained specialists. Advances in imaging techniques, novel antithrombotic and lipid lowering drugs, and endovascular therapies have increased the range of therapeutic options for patients with vascular diseases. In addition, vascular disorders require a distinct set of knowledge and skills for optimal management. This document provides the framework for training in this rapidly evolving field.

#### 1.2.2. Levels of Training

COCATS 4 updated standards for training fellows in Cardiovascular Medicine and established consistent training criteria across all aspects of general cardiology, including vascular medicine.^3^ For the cardiovascular fellowship, the following 3 levels of training are delineated for training in vascular medicine.

Level I training, the basic training required of trainees to become competent consultant cardiologists, is required of all fellows in cardiology, and can be accomplished as part of a standard 3-year training program in general cardiology. All cardiovascular fellows should receive basic training in vascular medicine and acquire sufficient knowledge to care for many patients with peripheral vascular diseases.

Level II training, also described in COCATS 4, refers to additional training that enables some cardiologists to perform or interpret specific procedures or render more specialized care for patients with certain conditions. Level II training in selected areas may be achieved by some trainees during the standard 3-year cardiovascular fellowship, depending on their career goals and use of elective rotations. In the case of vascular medicine, Level II training can be elected by fellows seeking additional expertise in interpreting noninvasive diagnostic tests and evaluating and managing patients with peripheral vascular diseases. Level II training is also recommended prior to or in conjunction with training in catheter-based peripheral vascular intervention (see COCATS 4 Task Force 10 report^5^).

Level III training, the primary focus of this document, requires training and experience beyond the cardiovascular fellowship for the acquisition of specialized knowledge and competency to render advanced care for patients with specific conditions. In the case of vascular medicine, Level III training pertains to advanced knowledge in diagnostic and therapeutic modalities for evaluating and managing vascular disease and leads to the ability to direct a vascular laboratory, train others, and conduct advanced research in vascular medicine.

## 2. General Standards

### 2.1. Faculty

Engaged faculty who are committed to teaching vascular medicine are critical to the success of an advanced vascular training program. Trainees should be exposed to individuals with advanced training in vascular medicine. In most institutions, the training program will be directed by a vascular medicine specialist who has met the Level III qualifications described in this document. In institutions where there is not a vascular medicine specialist, the training program may be coordinated by cardiologists, hematologists, neurologists, vascular surgeons, or vascular interventionalists. Key faculty members responsible for training fellows in vascular medicine should be board certified in their subspecialties. Vascular medicine training programs are encouraged to include faculty from relevant specialties, including vascular surgery, vascular radiology (diagnostic and interventional), hematology, neurology, dermatology, wound care, and rheumatology, which allows for a diverse and comprehensive training experience. Faculty should be committed to providing didactic and practical training spanning the topics of fundamental vascular pathophysiology; noninvasive and invasive diagnostic approaches; and the medical, interventional, and surgical management of patients with peripheral vascular disease. An emphasis on faculty who can support scholarly activity is encouraged, so that fellows can gain exposure to academic research.

### 2.2. Facilities

Training institutions should provide comprehensive facilities for the care of patients with vascular disease, including offices for outpatient evaluation and treatment as well as inpatient vascular consultative services. Institutions should also have an accredited noninvasive vascular laboratory and facilities for computed tomographic angiography, magnetic resonance (MR) angiography, and a safe and sterile environment for performing peripheral vascular catheterization and endovascular interventions. Comprehensive vascular surgery and wound care programs and facilities for supervised exercise rehabilitation should also be available.

### 2.3. Equipment

Noninvasive vascular laboratories require dedicated equipment to perform diagnostic studies, including duplex ultrasound units capable of high-resolution B-mode (grayscale) imaging as well as color and spectral Doppler analysis, equipment for physiological testing with appropriately sized cuffs to measure blood pressure at multiple sites in the limbs, Doppler and plethysmographic devices (eg, pulse volume recordings, photoplethysmographic sensors), and equipment for digital image recording and archiving. Equipment required for cardiovascular computed tomography (CT), cardiovascular MR, and catheter-based angiography is discussed in the COCATS 4 Task Force 7, 8, and 10 reports, respectively.^5–7^

### 2.4. Additional Resources

Level III trainees should communicate with additional personnel to ensure comprehensive, evidence-based care. Critical to success is collaborative interaction with various physicians from within the cardiovascular community, including vascular specialists, interventionalists, advanced imaging specialists, and other fields, in order to address and treat the multiple comorbidities often seen in patients with vascular disease. Key specialties include—but are not limited to—cardiac and vascular surgeons; nephrologists; hematologists; pulmonologists; neurologists; podiatrists; infectious disease specialists; cardiovascular genetic counselors; vascular sonographers; and experts in wound care, lymphatic decompression, exercise rehabilitation, prosthetics, and mobility aides. In addition, communication with primary care physicians, geriatricians, pediatricians, obstetricians, endocrinologists, and oncologists is important for specific patient populations. Also essential is interaction with other vital healthcare professionals, including nurse practitioners, physician assistants, nurses, pharmacists, dieticians, physical and occupational therapists, and social workers.

## 3. Training Components

### 3.1. Didactic Program

Didactic activities for Level III trainees should include a variety of formats, including lectures, online modules, journal clubs, grand rounds, clinical case presentations, research conferences, simulator-based training, and patient safety or quality improvement conferences. The educational content should cover vascular topics, including—but not limited to—peripheral artery disease; renal artery stenosis; mesenteric vascular disease; cerebrovascular disease; aneurysmal disease of the aorta and peripheral arteries; acute aortic syndromes; vasculitis; vasospastic and temperature-related diseases; venous thromboembolism; chronic venous insufficiency and varicose veins; lymphedema; less-common disorders such as fibromuscular dysplasia and arteriopathies associated with inherited diseases of connective tissue; congenital vascular malformations and arterial entrapment syndromes; vascular ulcers of the extremities; and the preoperative evaluation and perioperative management of patients undergoing vascular surgery. Educational content should also cover the noninvasive vascular laboratory, including principles of vascular physiology, ultrasound physics, vascular ultrasound imaging, Doppler flow velocity measurements, blood pressure measurement and pulse volume recordings, transducer technology, imaging artifacts, and the noninvasive evaluation of specific vascular diseases. Topics relevant to directing a noninvasive vascular laboratory should be covered, including budgeting, manpower, and equipment assessment, accreditation, RWI, sonographer supervision, and continuous quality improvement. Lectures and case presentations should cover other vascular imaging modalities such as MR, computed tomographic, and catheter-based angiography. The lecture series should include regularly scheduled patient safety or quality improvement conferences and journal clubs. There should be other forums for interactive discussion of the established literature and emerging scientific advances. Interaction with vascular specialists from other disciplines at these conferences is recommended.

### 3.2. Clinical Experience

Level III training in vascular medicine should provide the knowledge and skills to function as a vascular specialist, including the ability to interpret patients’ clinical presentation, plan diagnostic testing, apply clinical and laboratory information, and develop appropriate management plans for patients across the entire range of vascular diseases. This includes—but is not limited to— peripheral artery disease, renal artery disease, mesenteric vascular disease, cerebrovascular disease, aneurysmal disease of the aorta and peripheral arteries, acute aortic syndromes, vasculitis, venous thromboembolism, chronic venous insufficiency, varicose veins, lymphedema, extremity ulcers, vasospastic, temperature-related disorders, and less common vascular disorders. Trainees should acquire skills in the use of pharmacotherapy to prevent and treat atherosclerosis, venous thromboembolism, and associated risk factors; understand the roles of exercise rehabilitation and endovascular and surgical revascularization in management of patients with vascular disease; be capable of assessing cardiovascular risk; and be knowledgeable about periprocedural/perioperative management of patients undergoing endovascular procedures and vascular surgery. These skills should be obtained through patient engagement in ambulatory and inpatient settings.

Trainees should have dedicated time and experience in the noninvasive vascular laboratory to acquire skills in the performance and interpretation of noninvasive vascular tests, including physiological and ultrasound vascular testing, segmental pressure measurements, pulse volume recordings, and duplex ultrasonography, for venous thrombosis, venous insufficiency, peripheral artery disease, abdominal aortic aneurysm, renal and mesenteric artery disease, and cerebrovascular disease (see Section 3.3). The program also should ensure experience to acquire knowledge related to the other vascular diagnostic modalities (eg, MR, computed tomographic angiography, invasive angiography). Whereas Level III training in vascular medicine is not intended to provide the knowledge and skills to perform endovascular interventions, it is expected that trainees will have sufficient experience in a laboratory that performs diagnostic angiography and peripheral endovascular interventions to understand their use in the management of patients with vascular disease. It is also expected that trainees will have exposure to open vascular surgeries and the postoperative management of these patients.

### 3.3. Hands-On Procedural Experience

Level III trainees in vascular medicine will have acquired some hands-on procedural skills through prior Level I and II training, including skills to perform a detailed vascular physical examination, measurement of the ankle-brachial index, as well as additional skills as discussed in the COCATS 4 document.^4^ Advanced training in vascular medicine builds upon these previously acquired hands-on and procedural skills to include evaluation and management of rare and complex pathologies and development of skills for history taking and physical examination for these disorders. Building upon experience in measuring the ankle-brachial index, the Level III trainee should develop experience in using the handheld Doppler as an adjunct to the physical examination for bedside evaluation of extremity arteries and veins.

All Level III trainees should have extensive experience in the vascular laboratory in the interpretation of noninvasive vascular studies (see Section 4.2.8). Some trainees with prior Level II training in vascular medicine may have already certified as a Registered Physician in Vascular Interpretation (RPVI).^8^ During the advanced training program, time should be dedicated to acquisition of advanced interpretive skills, hands-on scanning exposure, and development of requisite skills to serve as the medical director of a noninvasive vascular laboratory. All Level III trainees should have significant hands-on experience in the performance of routine noninvasive vascular diagnostic studies, including carotid, renal, venous, mesenteric, aortic, and extremity arterial duplex ultrasonography, and hands-on experience in performing multilevel noninvasive physiological examinations at rest and with exercise. Such hands-on experience should include optimization of image acquisition. This hands-on experience in the vascular laboratory should be coordinated by an experienced and certified technologist/sonographer mentor (ie, registered vascular technologist [RVT] or equivalent credential) or a faculty mentor with extensive experience in vascular scanning as well as study interpretation. While it is not expected that Level III trainees will acquire adequate hands-on scanning experience and test volume to function independently as a technologist, it is important that Level III trainees have fundamental skills in performing noninvasive diagnostic tests in order to critically evaluate images for interpretation. These skills also help trainees address questions from the technologists in challenging cases and allow the Level III trainee to help train future technologists and medical staff when they start a vascular laboratory. Trainees may also acquire hands-on experience in management of post-catheterization complications during Level III training, including ultrasound-guided thrombin injection of post-catheterization pseudoaneurysms.

All Level III trainees will acquire hands-on skills in the management of swollen limbs due to chronic venous insufficiency and lymphedema as well as in the management of uncomplicated lower extremity wounds, including compression therapy management, indications for debridement, and dressing selection. With additional clinical rotations and hands-on experience, Level III trainees may acquire skills to manage complex lower extremity wounds, including care of wounds related to severe peripheral artery disease (PAD) (ie, critical limb ischemia) as part of a limb-salvage team, performance of mechanical debridement (when indicated), and use of adjunctive wound healing therapies (eg, negative pressure wound dressings). In addition to advanced wound care techniques, some individuals may pursue additional specialized training in procedural management of superficial venous disease during the vascular medicine fellowship, including hands-on training in sclerotherapy, chemical and thermal ablation, and ambulatory phlebectomy.

### 3.4. Diagnosis and Management of Emergencies and Complications

All trainees should know how to diagnose and manage acute vascular emergencies, including acute aortic syndromes, arterial dissection, acute limb ischemia, pulmonary embolism, acute venous thrombosis including phlegmasia cerulea dolens, complications of renal artery stenosis, acute mesenteric ischemia, transient ischemic attack or stroke in a patient with carotid artery disease, vascular access site complications, and bleeding complications of antithrombotic therapies. Often, management involves referral to other specialists, such as interventional cardiologists, interventional radiologists, or vascular surgeons, for advanced therapies.

### 3.5. Diagnosis and Management of Less Common Clinical Conditions and Syndromes

Vascular medicine trainees should be familiar with the less common vascular conditions they may encounter in their practice and when these conditions should be considered in the differential diagnosis of more common vascular diseases. These include—but are not limited to—vasculitis, fibromuscular dysplasia, arteriopathies associated with inherited diseases of connective tissue, entrapment syndromes, adventitial cystic disease, environmental and thermal-associated vascular diseases, and congenital vascular anomalies.

### 3.6. Research and Scholarly Activity

All trainees are encouraged to participate in research and scholarly activities during their vascular medicine fellowship. In some programs, these activities will occur concurrently with the clinical training; other programs will include an additional year or more of dedicated research. The scholarly activities can cover any topic related to vascular disease, including—but not limited to—atherosclerosis, thrombosis, aneurysm disease, venous/lymphatic disease, and vascular biology, and may include original basic, clinical, translational, population, health services, or health disparities research. Trainees are encouraged to present their work at regional and national meetings, and importantly are expected to disseminate their findings in high-quality, peer-reviewed journals. Case reports, book chapters, and review articles are also acceptable but should not replace original research. Trainees interested in academic careers in research should participate in activities geared toward acquiring skills to design, conduct, analyze, and interpret research as well as workshops or courses to enhance capabilities in grant and manuscript writing. All trainees are encouraged to develop and maintain habits of self-learning and continuous professional improvement through reading published literature, attending journal clubs and conferences, and participating in relevant scholarly meetings. A trainee’s progress in research should be monitored by the program director and the fellow’s faculty mentor, with metrics of success (eg, presentations, manuscripts, submitted grants) clearly specified at the beginning of the training program.

## 4. Training Requirements

### 4.1. Development and Evaluation of Core Competencies

Training requirements in vascular medicine address the 6 general competencies promulgated by the ACGME and endorsed by the ABIM. These competency domains are Medical Knowledge, Patient Care and Procedural Skills, Practice-Based Learning and Improvement, Systems-Based Practice, Interpersonal and Communication Skills, and Professionalism. The ACC has used this structure to define and depict the components of the clinical competencies for cardiovascular medicine. The curricular milestones for each competency and domain also provide a developmental roadmap for fellows as they progress through various levels of training and serve as an underpinning for the milestones reported to ACGME. The ACC has adopted this format for its competency and training statements, career milestones, lifelong learning, and educational programs.

Table 1 depicts the Medical Knowledge competencies and Patient Care and Procedural Skill competencies specifically related to vascular medicine, as well as examples of evaluation tools suitable for assessing competence in each domain. The focus of this document is on delineation of the core competencies expected of all trainees upon successful completion of a 1-year advanced vascular medicine fellowship. These competencies are marked under the column “all.” However, certain areas of advanced knowledge or procedural skills are not typically encountered enough during a standard 12-month period of training to develop and demonstrate competence and therefore require additional dedicated exposure. These selected Level III competency components are designated in the “add” column of Table 1, and usually require additional training. These additional competencies may be obtained during or after the standard vascular medicine fellowship, depending on the trainee’s career focus and the opportunities available at the training program. Although the competency components included in the “all” column should be achieved by all trainees and are appropriate areas for assessment, not every component need be individually assessed in every trainee. Rather, as with all educational activities, assessment is a sampling process that should be tailored to the needs of the individual trainee and program.

**Table 1.**
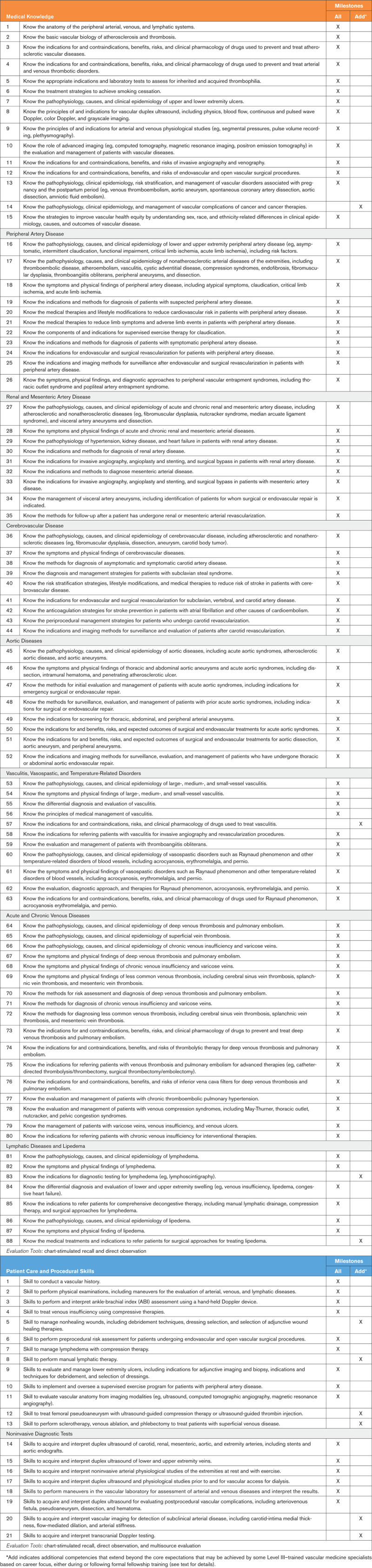
Competency Components and Curricular Milestones for Level III Training in Vascular Medicine

Table 2 specifies a common set of professional behavior competencies that fall under the ACGME competency domains entitled Systems-Based Practice, Practice-Based Learning and Improvement, Interpersonal and Communication Skills, and Professionalism. Although these competencies are relevant to all clinical cardiovascular disease specialists, they should be interpreted within the context of vascular medicine practice.

**Table 2.**
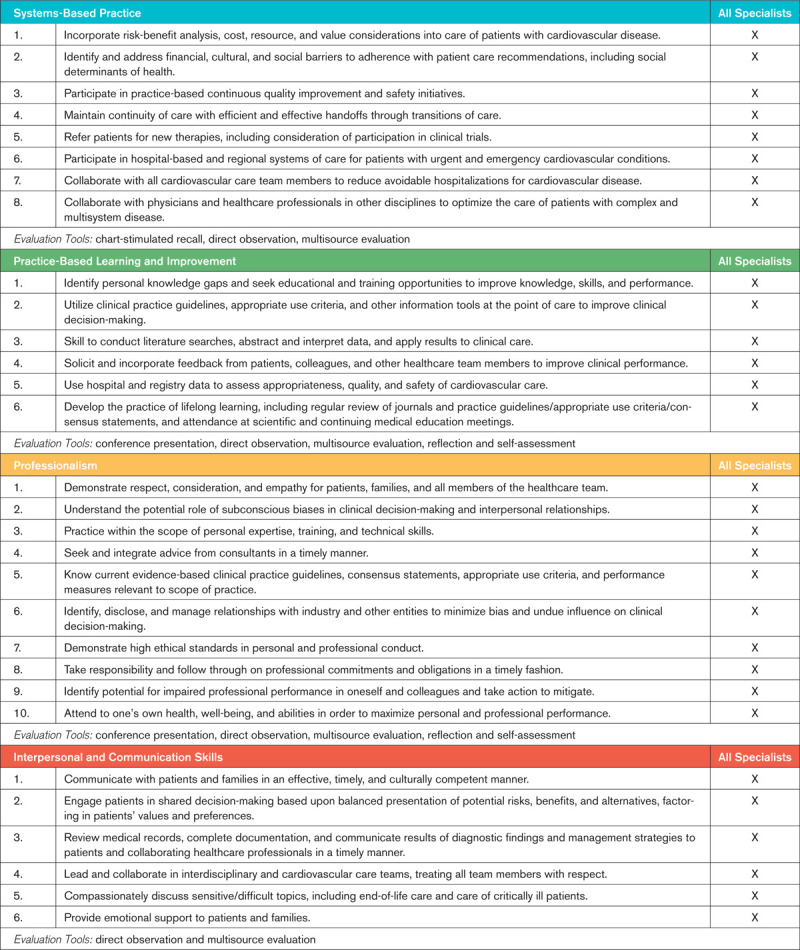
Common Professional Behavior Competencies to All Clinical Cardiovascular Disease Specialists

### 4.2. Vascular Medicine

Arterial, venous, and lymphatic disorders are common and induce substantial morbidity and mortality. The comprehensive management of all vascular disorders is the goal of Level III training in vascular medicine. All trainees should be able to manage acute and chronic vascular disorders in both the inpatient and outpatient setting, including initial evaluation and ongoing care. Core areas of knowledge include the anatomy, vascular biology, clinical pharmacology, epidemiology, pathophysiology, and treatment of diverse vascular diseases. In addition, vascular medicine trainees should understand the principles of vascular duplex and physiology testing and be familiar with the role of advanced imaging, angiography, and revascularization procedures.

#### 4.2.1. Peripheral Artery Disease

Given the growth of the aging population in the United States along with the known morbidity and mortality of PAD, it is crucial that vascular medicine trainees understand the pathophysiology, causes, epidemiology, risk factors, and symptoms of the disease such as claudication. In particular, trainees must be able to recognize the manifestations of the most severe forms of disease, acute and chronic critical limb ischemia, including pain in the limbs at rest, loss of lower extremity pulses, a nonhealing wound, or gangrene. PAD is diagnosed with the ankle-brachial index and toe brachial index.^9^ Trainees are expected to know the indications for different tests, how to perform them, and how to interpret them.^10^ Training requirements also include knowing the indications and limitations for the imaging modalities to diagnose these disorders (ie, duplex ultrasound, CT angiography, MR angiography, and catheter-based angiography). A trainee who has achieved competence in the vascular management of PAD should be knowledgeable about the current ACC/AHA recommendations for medical, endovascular, surgical, and exercise therapies and their application for the treatment of patients with PAD.^10^

#### 4.2.2. Renal and Mesenteric Artery Disease

It is important for trainees to understand the anatomy, physiology, and various disease states that may affect renal and visceral circulations. The most common manifestations are related to arterial stenosis secondary to atherosclerosis. The trainee should also be knowledgeable in other less common conditions such as fibromuscular dysplasia, renal and visceral artery aneurysms, dissection, vasculitis, and median arcuate ligament compression. Core training requirements also include knowing the indications and limitations for the imaging modalities to diagnose these disorders (ie, duplex ultrasound, CT angiography, MR angiography, and catheter-based angiography).

For patients with atherosclerotic renal artery disease and fibromuscular dysplasia, trainees should know the role of the renin-angiotensin-aldosterone axis in diagnosing and treating hypertension. They should also understand acute and chronic kidney disease states in these patients, as well as be cognizant of the presence of heart failure in patients with bilateral renal artery stenosis. The trainee must be familiar with optimal medical therapy for the management of patients with renal artery disease, as well as indications, risks and benefits of revascularization strategies. The trainee must also be familiar with the management of patients with mesenteric artery disease, including the risks and benefits of revascularization strategies. Trainees should know how to manage renal and visceral artery aneurysms and indications for referral for repair.

#### 4.2.3. Cerebrovascular Disease

Vascular medicine trainees must acquire the medical knowledge and clinical skills to diagnose and manage patients with extracranial cerebrovascular disease, including atherosclerotic and nonatherosclerotic diseases (such as fibromuscular dysplasia and carotid artery dissection). Core training requirements also include knowing the indications and limitations for the imaging modalities to diagnose cerebrovascular disease (ie, duplex ultrasound, CT angiography, MR angiography, and catheter-based angiography).

For patients with atherosclerotic cerebrovascular disease, trainees should define and apply appropriate risk stratification and medical therapies to reduce risk of stroke, including managing the most common risk factors for development and progression of cerebrovascular disease (eg, smoking, diabetes, hypertension, hypercholesterolemia). Trainees should also have a clear understanding of both the indications and risks associated with anticoagulation in patients with atrial fibrillation. Similarly, trainees should know the role of antiplatelet agents in prevention of stroke in patients with cerebrovascular disease.

Trainees should know the indications for intervention of carotid, vertebral, and subclavian artery stenosis. They should have broad exposure to the various surgical, endovascular, and hybrid interventions for management of cerebrovascular disease in order to recommend the optimal therapeutic strategy for patients in coordination with the care team.

Trainees should be capable of identifying early post-procedural complications (eg, stroke, hyperperfusion syndrome, cranial nerve injury) and the approach to management. They should be familiar with methods and intervals for long-term surveillance both prior to and after intervention.

#### 4.2.4. Aortic Diseases

Vascular medicine trainees will need to know the pathophysiology of a wide breadth of aortic diseases, including—but not limited to—acute aortic syndromes, aortic occlusive disease, aortic aneurysms. They should be knowledgeable about disorders associated with aortic aneurysm and dissection, such as inherited connective tissue disorders, familial thoracic aortic aneurysm syndromes, hypertension, bicuspid aortic valve, Turner’s syndrome, atherosclerosis, vasculitis, and infections. They should know the clinical signs and symptoms in patients presenting with high-risk aortic diseases such as aortic dissection, intramural hematoma, penetrating atherosclerotic ulcer, and thoracic and abdominal aneurysms. The ability to evaluate and medically manage acute aortic syndromes is necessary for competency. In addition, trainees will be expected to know the strategies necessary for the long-term follow-up of patients with prior acute aortic syndromes. Trainees should be knowledgeable regarding indications, accuracy, and techniques of screening for thoracic, abdominal, and peripheral arterial aneurysms. Once a diagnosis of acute or chronic aortic disease has been made, knowledge of the indications and risks of both surgical and endovascular treatment with expected outcomes are essential to provide appropriate care. Similarly, indications for surgical and endovascular repair of both aortic and peripheral aneurysms will be important for trainees. Trainees should be skilled at the longitudinal evaluation of patients who have undergone aortic aneurysm open and endovascular repair. When the diagnosis of aortic aneurysm is confirmed, trainees should be able to provide counseling and education to patients and their families on the importance of genetic predisposition and screening methods.

#### 4.2.5. Vasculitis, Vasospastic, and Temperature-Related Disorders

Vascular medicine trainees should learn when to consider vasculitis in the differential diagnosis of vascular disease and appropriately evaluate and diagnose small, medium, and large vessel vasculitis. Trainees should learn strategies for the medical management of vasculitis, including surveillance imaging, pharmacotherapy, and appropriate referral to or comanagement with rheumatology during the course of training. In addition, trainees should know when to refer patients for noninvasive and invasive diagnostic testing and revascularization. Similarly, trainees should acquire the knowledge and skills to diagnose, evaluate, and treat thromboangiitis obliterans; atheroembolism; erythromelalgia; and temperature-related disorders, including primary and secondary Raynaud phenomenon, acrocyanosis, erythema ab igne, and pernio.

#### 4.2.6. Acute and Chronic Venous Diseases

##### 4.2.6.1. Acute Venous Disease

Vascular medicine trainees should know the clinical epidemiology, pathophysiology, causes, and consequences of superficial and deep venous thrombosis and pulmonary embolism. Trainees should be familiar with the application of preventive strategies to reduce the incidence of venous thromboembolism and know the symptoms and signs of superficial and deep venous thrombosis pulmonary embolism, and the clinical risk stratification of patients with suspected venous thrombosis. Trainees should know appropriate use of biomarkers and imaging testing to diagnose venous thrombosis and pulmonary embolism and the acute management of venous thromboembolism, including clinical risk stratification and initial treatment regimens. They should know the appropriate indications and laboratory tests to assess for inherited and acquired thrombophilia. Trainees should also know the classes of anticoagulant medications and dosing regimens, as well as the benefits, risks, and complications of anticoagulant therapy, including those specific to each class of drugs. They should know the diagnostic and management strategies relevant to specific patient populations, such as pregnant women, patients with cancer, patients with severe obesity, and patients with chronic kidney disease. Trainees should be familiar with reversal agents used in the treatment of bleeding complications and know the indications and complications of inferior vena cava filters. Trainees should be familiar with advanced therapies, including the use of thrombolytic agents, and know the indications, contraindications, benefits, and risks for systemic and catheter-based thrombolysis, and catheter-based and surgical thrombectomy. Vascular medicine trainees must be knowledgeable about the causes, clinical presentation, and management of venous thrombosis of other anatomic locations, including inferior vena cava thrombosis, renal vein thrombosis, mesenteric venous thrombosis, portal vein thrombosis, and cerebral vein thrombosis.

##### 4.2.6.2. Chronic Venous Disease

Vascular medicine trainees should be familiar with the evaluation of chronic venous disease which comprises etiologies of post-thrombotic syndrome after initial or recurrent deep venous thrombosis; superficial venous disease; incompetent superficial, deep, or perforator disease; and compressive syndromes including iliac venous compression (May-Thurner syndrome) and venous thoracic outlet compression (Paget-Schroetter syndrome). Trainees should recognize the clinical symptoms of chronic venous disease such as pain, swelling, pruritus, and bleeding as well as the signs of chronic venous disease, including edema, varicose veins, skin changes of eczema, lipodermatosclerosis, corona phlebectatica, phlebolymphedema, and venous ulceration. Trainees should be able to classify severity using common scales such as the Venous Clinical Severity Scale (VCSS) and Clinical, Etiology, Anatomic, Pathophysiologic (CEAP) classification. Trainees should also be able to incorporate appropriate diagnostic tests, including duplex ultrasound to evaluate for obstruction and reflux, and MR and CT venograms to evaluate for obstruction or compressive syndromes. In addition, trainees should be familiar with the use of plethysmography to discern venous outflow obstruction and learn to manage patients with chronic venous disease using compression therapy—multilayer and short stretch bandaging, inelastic garments, graded compression stockings, and pneumatic pumps. Trainees should know the indications for the use of topical anti-inflammatory steroid medications as well as antibiotic and diuretic therapy. Trainees should be able to identify patients who would benefit from interventional endovascular therapies to address chronic venous obstruction and venous compression syndromes and know the signs and symptoms of pelvic congestion syndrome and indications to refer for further evaluation. Trainees should also be familiar with referral criteria for superficial venous ablation and the techniques available, including thermal ablation (radiofrequency and laser), ambulatory phlebectomy, and chemical ablation.

#### 4.2.7. Lymphatic Diseases and Lipedema

Vascular medicine trainees should know the pathophysiology of lymphedema and be able to identify patients at risk. Trainees should be able to establish a differential diagnosis of lymphedema, including lipedema, myxedema, venous insufficiency, and systemic diseases that cause leg swelling; develop clinical judgement to refer for the most appropriate diagnostic tests; and interpret the results. Trainees should be able to diagnose causes of primary and secondary lymphedema and identify lymphedema when associated with an underlying medical syndrome (eg, Turner syndrome, Noonan syndrome). They should be familiar with genetic variants associated with lymphedema and know when to refer for genetic evaluation and counseling. Using clinical examination and testing when appropriate, the trainee should be able to distinguish between lymphedema and lipedema and determine a management plan. When the diagnosis of lymphedema is confirmed, trainees should be able to provide counseling and education to the patient and family on the importance of compression and decongestive therapy, prevention and prompt treatment of infections, and skin care. They should be able to initiate compression therapy, refer to a physical therapist specialized in manual lymphatic therapy and multimodal lymphedema therapy, and determine if a patient would benefit from advanced therapies such as compression pumps or referral for surgery.

#### 4.2.8. Noninvasive Diagnostic Tests

Expert knowledge of noninvasive vascular diagnostic testing is a distinguishing feature of the Level III–trained vascular medicine specialist. The curriculum should include diagnostic testing procedures and equipment, appropriate indications, diagnostic criteria, and technical limitations. In addition to the curricular components, Level III trainees should have extensive mentored experience in interpreting vascular studies and have had significant hands-on exposure in vascular testing. The Physicians’ Vascular Interpretation Examination should be successfully completed after either Level II or III training. Interpretation under faculty supervision of at least 500 studies distributed across the vascular testing areas is a prerequisite for the Registered Physicians Vascular Interpretation Examination.^8^ The primary testing areas are duplex ultrasonography of the veins, extremity arteries (including bypass grafts and stents), extracranial (ie, carotid) and intracranial (ie, transcranial Doppler) arteries, the renal and mesenteric arteries, and the abdominal aorta as well as multilevel physiological testing of the extremities at rest and with exercise. As discussed in Section 3.3, it is acknowledged that some trainees who have completed Level II training in vascular medicine prior to the advanced training program will have already obtained adequate experience to qualify for the RPVI examination. For such individuals, and all Level III trainees, additional time spent in the vascular laboratory during the training program should focus on acquisition of hands-on scanning skills (Section 3.3) as well as skills to interpret and perform ultrasound assessment and treatment of post-catheterization access site complications (ie, pseudoaneurysm, arteriovenous fistula). Additional training is necessary to perform and interpret transcranial Doppler tests for assessment of intracranial arteries and perform and analyze vascular tests used for research (eg, carotid intimal-medial thickness, brachial reactivity, arterial stiffness assessment). For Level III trainees with future interest in serving as Medical Director of a noninvasive vascular laboratory, additional didactic topics and experience should include: principles of appropriateness of vascular testing, developing a quality-improvement program, including processes for peer review and cross-modality correlative imaging study, vascular laboratory accreditation, and operational aspects of the vascular laboratory, including equipment utilization and maintenance. As training in the vascular laboratory is a central component of advanced training in vascular medicine, it is important that comprehensive facilities and dedicated equipment are available and that laboratories are accredited by the Intersocietal Accreditation Commission Vascular Testing Division.^11^ Faculty members, including allied health professionals, should have extensive experience in noninvasive vascular testing, including certification of physician faculty and technologists/sonographers by the RPVI, RVT, or equivalent discipline-appropriate credential.

## 5. Leadership and Administrative Competencies

In addition to clinical competency, Level III–trained vascular specialists are expected to function effectively as leaders in allied efforts to ensure high-quality care and promote individual and population health. Some of these activities and attributes fall outside the realm of clinical knowledge and skill and instead involve administrative roles in clinical practice, hospitals, health systems, professional societies, or other organizations. Developing a familiarity with accreditation standards for vascular laboratories and participation in an accreditation process are examples of how an advanced vascular medicine fellow can gain experience in laboratory administration.^11^ The intention of training is to provide a foundation of leadership and administrative skills that would be enhanced and refined throughout one’s career after fellowship. Specific competencies expected of all general cardiologists and cardiovascular specialists, including those whose careers involve greater involvement in administrative, managerial, or advocacy positions, are delineated in Table 18 of the 2016 ACC Lifelong Learning Competencies for General Cardiologists.^2^

## 6. Evaluation of Proficiency

Evaluation of a trainee’s proficiency in advanced training in vascular medicine involves multiple assessments of the trainee’s ability to function as a specialist to clinically diagnose and manage patients across the broad range of vascular disease; apply clinical and laboratory information; and plan, perform, and accurately interpret vascular diagnostic studies. These assessments include multisource evaluation, direct observation by instructors, case logs, chart review (including adherence to utilization guidelines, appropriate use criteria, and patient outcomes), simulation training, the trainee’s portfolio (including scholarly productivity and quality improvement projects), and assessment of leadership skills. The trainee’s organization of, and participation in, didactic conferences and case presentations also provides opportunities to evaluate the trainee’s proficiency. Trainees have access to self-assessment programs through the ACC and the SVM.

Each trainee must be evaluated regularly regarding clinical judgment, case management, and procedural skills. This includes consideration of judgments or actions that may result in complications; quality of care and follow-up management; interactive behaviors with patients and their families and other members of the health care team; and ability to independently make appropriate decisions. Trainees must maintain records of participation and advancement using an electronic database or procedure logbook that is Health Insurance Portability and Accountability Act (HIPAA) compliant and contains pertinent clinical information, including number of cases, diagnoses (including comparison of vascular study interpretation with other available vascular diagnostic modalities), disease severity, outcomes, and disposition.

The vascular medicine training program director is responsible for confirming trainee experience and competence, working with faculty instructors to verify and document trainee performance, and reviewing the overall progress of trainees to ensure achievement of training milestones and identification of areas in which additional focused training may be required. On a periodic basis, the program director should review each trainee’s case logbook to ensure adequacy of exposure to a broad spectrum of pathology and experience in acquiring and interpreting diagnostic vascular studies.

Following the completion of advanced training in vascular medicine, trainees are expected to take the certification examination in vascular medicine offered by the American Board of Vascular Medicine (ABVM). Information concerning eligibility and prerequisites for this certification can be obtained from ABVM.^12^ Additionally, trainees are expected to have completed certification for interpreting noninvasive vascular examinations such as the Registered Physician in Vascular Interpretation (RPVI) credential offered by the Alliance for Physician Certification and Advancement (APCA).^8^ For trainees who have completed supplemental training in performance of vascular studies during their fellowship, the American Registry for Diagnostic Medical Sonography (ARDMS) provides additional certification with the Registered Vascular Technologist (RVT) credential offered by the ARDMS.^13^ Information concerning eligibility and prerequisites for these certifications can be obtained from APCA and ARDMS.

## ACC Competency Management Committee

James A. Arrighi, MD, FACC, Chair; Lisa A. Mendes, MD, FACC, Co-Chair; Jennifer Day, MSN, RN; G. William Dec Jr, MD, FACC; Ali Denktas, MD, FACC; David Drajpuch, ACNP-BC, CRNP, FNP-BC, MSN; Susan Fernandes, LPD, PA-C*; Sanjeev A. Francis, MD, FACC; Rebecca T. Hahn, MD, FACC; Susan D. Housholder-Hughes, DNP, ACNS-BC, ANP-BC, FACC; Sadiya S. Khan, MD, FACC; Kyle Klarich, MD, FACC*; Meera Devi Kondapaneni, MBBS, FACC; Kwan S. Lee, MD, FACC; C. Huie Lin, MD, PhD, FACC; Joseph E. Marine, MD, FACC*; Shannon McConnaughey, MD, FACC; Khusrow Niazi, MBBS, FACC; Thomas Ryan, MD, FACC; Frank E. Silvestry, MD, FACC; Michael A. Solomon, MD, MBA, FACC; Robert L. Spicer, MD, FACC; Andrew Wang, MD, FACC, FAHA*; Gaby Weissman, MD, FACC; Howard H. Weitz, MD, MACP, FACC*

## Presidents and Staff

### American College of Cardiology

Athena Poppas, MD, FACC, President Cathleen C. Gates, Chief Executive Officer Janice Sibley, MS, Executive Vice President, Education and Publishing Dawn R. Phoubandith, MSW, Team Leader, Competencies and Educational Gaps Teresa V. Callahan, Document Production Specialist Grace Ronan, Team Lead, Clinical Policy Publication

### American Heart Association

Mitchell S.V. Elkind, MD, MS, FAAN, FAHA, President Nancy Brown, Chief Executive Officer Mariell Jessup, MD, FAHA, Chief Science and Medical Officer

### Society for Vascular Medicine

Raghu Kolluri, MS, MD, FSVM, President Emily Burch, Executive Director

### American College of Physicians

Jacqueline Winfield Fincher, MD, MACP, President Darilyn V. Moyer, MD, FACP, FRCP, FIDSA, Chief Executive Officer, Executive Vice President Davoren Chick, MD, FACP, Senior Vice President, Medical Education

## Supplementary Material



## Footnotes

* Former Competency Management Committee member; member during this writing effort.

## References

[1] Alpert JS. Guidelines for training in adult cardiovascular medicine core cardiology training symposium (COCATS) June 27–28, 1994. J Am Coll Cardiol. 1995;25:1–2. 7798524 10.1016/0735-1097(95)96215-k

[2] Williams ES Halperin JL Arrighi JA. 2016 ACC lifelong learning competencies for general cardiologists: a report of the ACC Competency Management Committee. J Am Coll Cardiol. 2016;67:2656–95. 26902874 10.1016/j.jacc.2016.02.011

[3] Halperin JL Williams ES Fuster V. COCATS 4 introduction. J Am Coll Cardiol. 2015;65:1724–33. 25777643 10.1016/j.jacc.2015.03.020

[4] Creager MA Gornik HL Gray BH. COCATS 4 task force 9: training in vascular medicine. J Am Coll Cardiol. 2015;65:1832–43. 25777653 10.1016/j.jacc.2015.03.025

[5] King SB Babb JD Bates ER. COCATS 4 task force 10: training in cardiac catheterization. J Am Coll Cardiol. 2015;65:1844–53. 25777641 10.1016/j.jacc.2015.03.026

[6] Garcia MJ Blankstein R Budoff MJ. COCATS 4 task force 7: training in cardiovascular computed tomographic imaging. J Am Coll Cardiol. 2015;65:1810–21. 25777650 10.1016/j.jacc.2015.03.028

[7] Kramer CM Hundley WG Kwong RY. COCATS 4 task force 8: training in cardiovascular magnetic resonance imaging. J Am Coll Cardiol. 2015;65:1822–31. 25777642 10.1016/j.jacc.2015.03.022

[8] Alliance for Physician Certification and Advancement (APCA). Available at: https://www.apca.org/certifications-examinations/registered-physician-in-vascular-interpretation/physicians-vascular-interpretation-pvi/. Accessed January 10, 2020.

[9] Aboyans V Criqui MH Abraham P. Measurement and interpretation of the ankle-brachial index: a scientific statement from the American Heart Association. Circulation. 2012;126:2890–909. 23159553 10.1161/CIR.0b013e318276fbcb

[10] Gerhard-Herman MD Gornik HL Barrett C. 2016 AHA/ACC guideline on the management of patients with lower extremity peripheral artery disease: a report of the American College of Cardiology/American Heart Association task force on clinical practice guidelines. Circulation. 2017;135:e686–725. 27840332 10.1161/CIR.0000000000000470 PMC5479414

[11] Intersocietal Accreditation Commission. IAC standards and guidelines for vascular testing accreditation. Available at: https://www.intersocietal.org/vascular/standards/IACVascularTestingStandards2019.pdf. Accessed October 30, 2019.

[12] American Board of Vascular Medicine (ABVM) certification requirements. Available at: https://www.vascularboard.org/cert_reqs.cfm. Accessed January 10, 2020.

[13] American Registry of Diagnostic Medical Sonography (ARDMS) certification requirements for the Registered Vascular Technologist (RVT). Available at: https://www.ardms.org/get-certified/rvt/vascular-technology/. Accessed January 10, 2020.

