# Supplementary material for: 2021 ACC/AHA/SVM/ACP Advanced Training Statement on Vascular Medicine (Revision of the 2004 ACC/ACP/SCAI/SVMB/SVS Clinical Competence Statement on Vascular Medicine and Catheter-Based Peripheral Vascular Interventions)
Source: Circ Cardiovasc Interv. 2021 Jan 15;14(2):e000079. doi: 10.1161/HCV.0000000000000079 (PMC8221116; doi:10.1161/HCV.0000000000000079)
Supplement: Supplementary file 1 [file hcv-14-e000079-s001.pdf]

AUTHOR RELATIONSHIPS WITH INDUSTRY AND OTHER ENTITIES (COMPREHENSIVE)—2021 ACC/AHA/SVM/ACP ADVANCED TRAINING STATEMENT ON VASCULAR MEDICINE

Committee Member	Employment	Consultant	Speakers Bureau	Ownership/ Partnership/ Principal	Personal Research	Institutional/ Organizational or Other Financial Benefit	Expert Witness
Mark A. Creager *(Chair)*	Dartmouth-Hitchcock Medical Center—Director, Heart and Vascular Center	None	None	None	None	•World Heart Federation*	None
Naomi M. Hamburg *(Vice Chair)*	Boston University School of Medicine—Associate Professor of Medicine, Cardiovascular Medicine Section	•Amgen<br>•Bayer<br>•Merck<br>•Sanifit	None	None	•NIH†	None	None
Keith D. Calligaro	Pennsylvania Hospital—Chief, Vascular Surgery; Director Vascular Surgery Fellowship; Clinical Professor of Surgery	None	None	None	None	None	• Defendant, Mumford vs. Abington Hospital, 2019<br>• Plaintiff, Sintef vs. Baubitz, 2019<br>• Defendant, Zebroski vs. Mawn, 2019†<br>• Defendant, Robinson vs. Lewis, 2019<br>• Defendant, Cobaugh vs. Amin, 2019<br>• Defendant, Broebeck vs. LVH, 2019†<br>• Defendant, Miller vs. Dubois Medical Center, 2018†<br>• Defendant, Melton vs. Geisinger, 2018†<br>• Defendant, Harmon vs. Shah, 2018†<br>• Defendant, Sandorha vs. Sandu, 2018<br>• Defendant, Isiah vs. Abai, 2018<br>• Defendant, Stanford vs. Einstein, 2018

© 2021 by the American College of Cardiology Foundation, the American Heart Association, Inc., and Society for Vascular Medicine.

Committee Member	Employment	Consultant	Speakers Bureau	Ownership/ Partnership/ Principal	Personal Research	Institutional/ Organizational or Other Financial Benefit	Expert Witness
Ana I. Casanegra	Mayo Clinic Gonda Vascular Center—Director of the Thrombosis and Thrombophilia Clinic; Mayo Clinic, Cardiovascular Department, Vascular Medicine Division—Associate Professor of Medicine	None	None	None	None	None	None
Rosario Freeman	University of Washington—Professor of Medicine; Program Director, Cardiology Fellowship	None	None	None	None	None	None
Phyllis A. Gordon	University of Texas Health Science Center at San Antonio—Vascular Surgery Division Assistant Clinical Professor (Retired)	None	None	None	None	None	None
Heather L. Gornik	University Hospitals Harrington Heart and Vascular Institute—Co-Director, Vascular Center; Case Western Reserve University School of Medicine—Professor of Medicine	•Northwestern University	None	•Flexlife Health†	None	•Fibromuscular Dysplasia Society of America*<br>•IAC - Vascular Testing*<br>•Society for Vascular Medicine*	None
Esther S.H. Kim	Vanderbilt Heart and Vascular Institute—Associate Professor of Medicine Cardiovascular Medicine Section of Vascular Medicine	None	None	None	None	•Acer Pharmaceuticals	None
Nicholas J. Leeper	Stanford University School of Medicine—Chief of Vascular Medicine, Director of Vascular Research, Professor of Surgery and Medicine	•Forty Seven<br>•Janssen Pharmaceuticals†<br>•Sanofi	None	None	•Bayer‡	None	None

© 2021 by the American College of Cardiology Foundation, the American Heart Association, Inc., and Society for Vascular Medicine.

Committee Member	Employment	Consultant	Speakers Bureau	Ownership/ Partnership/ Principal	Personal Research	Institutional/ Organizational or Other Financial Benefit	Expert Witness
Geno J. Merli	Thomas Jefferson University Hospital—Sr. Vice President, Associate CMO; Jefferson Vascular Center—Co-Director; Sidney Medical College—Professor of Medicine and Surgery, Division of Vascular Medicine	•LoweRisk*	None	None	None	None	None
Khusrow Niazi	Emory University—Director, Peripheral Vascular Intervention	None	•Medtronic	None	•Surmodics† •Trireme†	•Surmodics‡ •Trireme‡	None
Jeffrey W. Olin	Zena and Michael A. Wiener Cardiovascular Institute and Marie-Josée and Henry R. Kravis Center for Cardiovascular Health Icahn School of Medicine at Mount Sinai—Professor of Medicine (Cardiology); Director, Vascular Medicine and Vascular Diagnostic Laboratory	•Fibromuscular Dysplasia Society of America* •Janssen Pharmaceuticals	None	None	None	None	None
Rene Quiroz	Cardiology Clinic of San Antonio—Cardiologist	None	None	•Wellvana†	None	•Medtronic‡	•Defendant, anoxic brain injury, 2018
Elona Rrapo Kaso	University of Virginia Health System—Fellow in Advanced Cardiovascular Imaging, Division of Cardiovascular Medicine	None	None	None	None	None	None
Suman Wasan	University of North Carolina—Rex Healthcare Vascular Specialists, Raleigh	•Diagnostica Stago •Janssen Pharmaceuticals •Tactile	None	•ABVLM* •CCI*	•Diagnostica Stago† •University of Washington	None	None

© 2021 by the American College of Cardiology Foundation, the American Heart Association, Inc., and Society for Vascular Medicine.

Committee Member	Employment	Consultant	Speakers Bureau	Ownership/ Partnership/ Principal	Personal Research	Institutional/ Organizational or Other Financial Benefit	Expert Witness
Andrew R. Waxler	Pennsylvania State College of Medicine—Clinical Assistant Professor of Medicine; Philadelphia College of Osteopathic Medicine—Clinical Assistant Professor of Medicine	None	•Amarin† •CardioDx •Sanofi, Regeneron† •ZOLL Medical†	None	None	•DalCor‡ •The Medicines Company‡ •Pfizer‡ •Sanofi, Regeneron	None
Christopher J. White	Ochsner Medical Center—Professor and Chairman of Medicine & Cardiology; Medical Director Value Based Care System Chair for Cardiovascular Diseases	None	None	None	None	•NIH	None
Khendi White Solaru	University Hospitals Harrington Heart and Vascular Institute—Assistant Professor, Department of Medicine; Case Western Reserve University School of Medicine Department of Cardiovascular Medicine—Staff Physician	None	None	None	None	None	None
Marlene S. Williams	The Johns Hopkins University—Associate Professor of Medicine; Johns Hopkins Bayview Medical Center—Cardiac Intensive Care Unit Director	•Haemonetics	None	None	None	None	None

This table represents all healthcare relationships of committee members with industry and other entities by authors, including those not deemed to be relevant, at the time this document was under development. The table does not necessarily reflect relationships with industry at the time of publication. A person is deemed to have a significant interest in a business if the interest represents ownership of ≥5% of the voting stock or share of the business entity, or ownership of ≥$ 5,000 of the fair market value of the business entity; or if funds received by the person from the business entity exceed 5% of the person's gross income for the previous year. Relationships that exist with no financial benefit are also included for the purpose of transparency. Relationships in this table are modest unless otherwise noted. Please refer to http://www.acc.org/guidelines/about-guidelines-and-clinical-documents/relationships-with-industry-policy for definitions of disclosure categories or additional information about the ACCF Disclosure Policy for Writing Committees.

*No financial benefit.
†Significant relationship.
‡This disclosure was entered under the Clinical Trial Enroller category in the ACC's disclosure system. To appear in this category, the author acknowledges that there is no *direct* or *institutional* relationship with the trial sponsor, as defined in the ACCF Disclosure Policy for Document Development.

© 2021 by the American College of Cardiology Foundation, the American Heart Association, Inc., and Society for Vascular Medicine.

ABVLM indicates American Board of Venous and Lymphatic Medicine; ACC, American College of Cardiology; ACP, American College of Physicians; AHA, American Heart Association; BMS, Bristol-Myers Squibb; CCI, Cardiovascular Credentialing International; IAC, Intersocietal Accreditation Commission; NIH, National Institutes of Health; and SVM, Society for Vascular Medicine.

© 2021 by the American College of Cardiology Foundation, the American Heart Association, Inc., and Society for Vascular Medicine.
