# Supplementary material for: 2021 ACC/AHA/SVM/ACP Advanced Training Statement on Vascular Medicine (Revision of the 2004 ACC/ACP/SCAI/SVMB/SVS Clinical Competence Statement on Vascular Medicine and Catheter-Based Peripheral Vascular Interventions)
Source: Circ Cardiovasc Interv. 2021 Jan 15;14(2):e000079. doi: 10.1161/HCV.0000000000000079 (PMC8221116; doi:10.1161/HCV.0000000000000079)
Supplement: Supplementary file 2 [file hcv-14-e000079-s002.pdf]

REVIEWER RELATIONSHIPS WITH INDUSTRY AND OTHER ENTITIES (COMPREHENSIVE)—2021 ACC/AHA/SVM/ACP ADVANCED TRAINING STATEMENT ON VASCULAR MEDICINE

Name	Employment	Representation	Consultant	Speakers Bureau	Ownership/ Partnership/ Principal	Personal Research	Institutional/ Organizational or Other Financial Benefit	Expert Witness
Geoffrey D. Barnes	University of Michigan—Assistant Professor of Internal Medicine	Official Reviewer, SVM	•AMAG<br>•BMS/Pfizer*<br>•Janssen Pharmaceuticals<br>•Portola*	None	None	•Blue Cross Blue Shield of Michigan*<br>•BMS/Pfizer*	None	None
Joshua A. Beckman	Vanderbilt University Medical Center—Director, Vascular Medicine Section; Professor of Medicine	Official Reviewer, SVM	•Amgen<br>•AstraZeneca*<br>•JanOne<br>•Sanofi*	None	•EMX†<br>•JanaCare†	•Bayer (DSMB)<br>•Novartis (DSMB)	•Vascular Interventional Advances*	None
Umberto Campia	Brigham and Women's Hospital—Associate Physician	Official Reviewer, AHA	None	None	None	None	None	None
Douglas E. Drachman	Massachusetts General Hospital—Heart Center Director of Education	Official Reviewer, ACC Lifelong Learning Oversight Committee	•Abbott Vascular*<br>•Boston Scientific*<br>•Broadview Ventures<br>•CSI*	None	None	None	•Atrium Medical<br>•Lutonix/Bard	None
Natalie S. Evans	Case Western Reserve University School of Medicine—Clinical Assistant Professor	Official Reviewer, AHA	None	None	None	None	•Amgen	None

© 2021 by the American College of Cardiology Foundation, the American Heart Association, Inc., and Society for Vascular Medicine.

Name	Employment	Representation	Consultant	Speakers Bureau	Ownership/ Partnership/ Principal	Personal Research	Institutional/ Organizational or Other Financial Benefit	Expert Witness
Sanjeev A. Francis	Maine Medical Center MaineHealth Cardiology—Director of Education, Cardiovascular Institute Director, Cardiovascular Medicine Fellowship Program; Tufts University School of Medicine—Assistant Professor of Medicine	Official Reviewer, Competency Management Committee Lead Reviewer	None	None	None	None	None	None
Sanjay Gandhi	MetroHealth Hospital—Medical Director of Hospital Strategy and Innovation; Case Western University—Associate Professor of Medicine	Official Reviewer, ACC Board of Governors	None	None	None	•Athersys (DSMB)<br>•Cleveland HeartLab<br>•Juventas Therapeutics<br>•Tendyne	•Juventas Therapeutics	None
James Kumar	Carle Foundation Hospital, University of Illinois—Clinical Associate Professor, Director, Carle Internal Medicine Residency Program	Official Reviewer, ACP	None	None	None	None	None	None
Aruna Pradhan	Brigham and Women's Hospital—Associate Physician; Harvard Medical School—Associate Professor of Medicine	Official Reviewer, AHA	•Optum Health*	None	None	•Denka-Seiken*<br>•Kowa Research Institute*	None	None

© 2021 by the American College of Cardiology Foundation, the American Heart Association, Inc., and Society for Vascular Medicine.

Name	Employment	Representation	Consultant	Speakers Bureau	Ownership/ Partnership/ Principal	Personal Research	Institutional/ Organizational or Other Financial Benefit	Expert Witness
Eric A. Secemsky	Beth Israel Deaconess Medical Center—Director of Vascular Intervention; Harvard Medical School—Assistant Professor of Medicine	Official Reviewer, SVM	•CSI*<br>•Medtronic*<br>•Philips	•BD Bard<br>•Cook*	None	•Astra Zeneca*<br>•BD Bard*<br>•Boston Scientific*<br>•Cook*<br>•CSI*<br>•Medtronic*<br>•Philips*	None	None
Herbert D. Aronow	Lifespan Cardiovascular Institute—Director, Interventional Cardiology; Rhode Island & The Miriam Hospitals—Director, Cardiac Catheterization Laboratories; Alpert Medical School of Brown University—Associate Professor of Medicine	Organizational Reviewer, SCAI; Content Reviewer, ACC Interventional Section Leadership Council	•Silk Road Medical	None	None	•Philips (DSMB)	•CSI<br>•Medtronic<br>•NIH<br>•SCAI†<br>•Shockwave Medical<br>•SVM (Board of Trustees)†	None
Jasmiry Bennett	Baylor Heart and Vascular Hospital—Advance Practice Professional Manager	Organizational Reviewer, SVN	None	None	None	None	None	None
John M. Fontaine	Drexel University College of Medicine—Professor of Medicine	Organizational Reviewer, ABC	None	None	None	None	None	None
Peter Henke	University of Michigan—Professor of Surgery	Organizational Reviewer, SVS	None	None	None	None	None	•Defendant, renal artery aneurysm rupture, 2019

© 2021 by the American College of Cardiology Foundation, the American Heart Association, Inc., and Society for Vascular Medicine.

Name	Employment	Representation	Consultant	Speakers Bureau	Ownership/ Partnership/ Principal	Personal Research	Institutional/ Organizational or Other Financial Benefit	Expert Witness
Uzoma N. Ibebuogu	University of Tennessee Health Science Center—Associate Professor of Medicine, Cardiology; Associate Professor of Preventive Medicine	Organizational Reviewer, ABC	None	None	None	None	None	None
Sasanka Jayasuriya	Ascension Columbia St. Mary's Hospital—Director Cardiac Catheterization Laboratories	Organizational Reviewer, SCAI	None	None	None	None	None	None
Ashley Moore	Texas Vascular Associates—Adult-Gerontology Acute Care Nurse Practitioner.	Organizational Reviewer, SVN	None	None	None	None	None	None
Thomas F. Rehring	Colorado Permanente Medical Group—Chief, Vascular and Endovascular Surgery	Organizational Reviewer, SVS	None	None	None	None	None	None
Subhash Banerjee	University of Texas Southwestern Medical Center—Professor of Medicine; VA North Texas Health Care System—Chief, Division of Cardiology & Director Cardiac Catheterization Laboratory	Content Reviewer, ACC Peripheral Vascular Disease Section Leadership Council	•LIVMOR*	•Astra Zeneca*<br>•Medtronic*	None	•Boston Scientific*	•Cardiovascular Innovations Foundations*<br>•CSI<br>•Philips	None

© 2021 by the American College of Cardiology Foundation, the American Heart Association, Inc., and Society for Vascular Medicine.

Name	Employment	Representation	Consultant	Speakers Bureau	Ownership/ Partnership/ Principal	Personal Research	Institutional/ Organizational or Other Financial Benefit	Expert Witness
Sunit-Preet Chaudhry	St. Vincent Indianapolis, St. Vincent Heart Center—Advanced Heart Failure and Transplant Cardiologist	Content Reviewers, ACC Early Career Section Leadership Council	None	None	None	None	None	None
G. William Dec	Massachusetts General Hospital—Chief (Emeritus), Cardiology Division	Content Reviewer, Competency Management Expertise	None	None	None	• Theracos (DSMB)	None	None
Kim A. Eagle	University of Michigan School of Public Health—Albion Walter Hewlett Professor of Internal Medicine, Professor of Health Management and Policy; Frankel Cardiovascular Center at the University of Michigan—Director	Content Reviewer, ACC Lifelong Learning Oversight Committee	None	None	None	• GORE, Medtronic	None	None
Robert T. Eberhardt	Boston University School of Medicine—Associate Professor of Medicine in Cardiovascular Medicine and Surgery in Endovascular and Vascular Surgery	Content Reviewer, ACC Peripheral Vascular Disease Section Leadership Council	None	None	None	None	None	None
Marci Farquhar-Snow	Mayo Clinic—Cardiovascular Department, Assistant Professor of Medicine	Content Reviewer, ACC CV Team Section Leadership Council	None	None	None	None	None	None

© 2021 by the American College of Cardiology Foundation, the American Heart Association, Inc., and Society for Vascular Medicine.

Name	Employment	Representation	Consultant	Speakers Bureau	Ownership/ Partnership/ Principal	Personal Research	Institutional/ Organizational or Other Financial Benefit	Expert Witness
Lawrence A. Garcia	Steward St. Elizabeth's Medical Center—Chief, Interventional Cardiology and Vascular Interventions	Content Reviewer, ACC Peripheral Vascular Disease Section Leadership Council	•Boston Scientific† •Pathway Medical/BSC†	None	•Arsenal Medical •CVI* •Essential Medical* •Orchestra* •Primacea* •Scion Cardiovascular* •Syntervention* •TissueGen* •Transit Scientific* •Innovation Vascular Partners*	•BSC† •ev3/Covidien /Medtronic* •iDev/Abbott*	•NIH •JET-RANGER	None
Bruce H. Gray	Prisma Health System, Greenville Memorial Hospital—Vascular Medicine Specialist; University of South Carolina School of Medicine—Professor of Surgery and Vascular Medicine	Content Reviewer, COCATS 4 Task Force 9 Writing Committee Member	None	None	None	None	•Cordis •Gore	None
Katie Greenlee	Cleveland Clinic—Cardiology Clinical Specialist	Content Reviewer, ACC CV Team Section Leadership Council	None	None	None	None	None	None

© 2021 by the American College of Cardiology Foundation, the American Heart Association, Inc., and Society for Vascular Medicine.

Name	Employment	Representation	Consultant	Speakers Bureau	Ownership/ Partnership/ Principal	Personal Research	Institutional/ Organizational or Other Financial Benefit	Expert Witness
T. Sloane Guy	Thomas Jefferson University Hospital—Professor of Surgery	Content Reviewer, ACC Surgeons Section Leadership Council	• Edward Lifesciences<br>• Ethicon, Johnson & Johnson Medtronic	None	None	None	Intuitive Surgical†	None
Deborah Hornacek	Cleveland Clinic, Tomsich Family Department of Cardiovascular Medicine—Staff physician, Section of Vascular Medicine; Program Director of Vascular Medicine Fellowship; Cleveland Clinic Lerner College of Medicine of Case Western Reserve University—Clinical Assistant Professor of Medicine	Content Reviewer	None	None	None	None	None	None
Susan D. Housholder-Hughes	University of Michigan—Nurse Practitioner, Adjunct Clinical Faculty, School of Nursing	Content Reviewer, Competency Management Expertise	None	None	None	None	None	None
Richard A Josephson	Case Western Reserve University—Professor of Medicine; Harrington Heart & Vascular Institute, University Hospitals—Director of Cardiovascular and	Content Reviewer, Prevention of Cardiovascular Disease Section Leadership Council	None	None	None	None	None	None

© 2021 by the American College of Cardiology Foundation, the American Heart Association, Inc., and Society for Vascular Medicine.

Name	Employment	Representation	Consultant	Speakers Bureau	Ownership/ Partnership/ Principal	Personal Research	Institutional/ Organizational or Other Financial Benefit	Expert Witness
	Pulmonary Rehabilitation							
Scott Kinlay	VA Boston Healthcare System—Associate Chief Cardiology and Director of Vascular Medicine	Content Reviewer, Peripheral Vascular Disease Section Leadership Council	None	None	None	•Colorado Prevention Center (DSMB)	•American Board of Vascular Medicine†	None
Viet T. Le	Intermountain Heart Institute, Intermountain Healthcare and Rocky Mountain University of Health Professions Physician Assistant Program—Cardiology Research Physician Assistant	Content Reviewer, ACC CV Team Section Leadership Council	None	None	None	None	None	None
Radmila Lyubarova	Albany Medical Center—Associate Professor of Medicine Division of Cardiology	Content Reviewer, ACC Geriatric Section Leadership Council	None	None	None	None	None	None
Carlos Mena	Yale New Haven Hospital—Director, Cardiac Catheterization Laboratories and Director, Vascular Medicine Fellowship; Yale University—Associate Professor of Medicine, Cardiology	Content Reviewer, ACC Peripheral Vascular Disease Section Leadership Council	•Abbott* •Bard •Boston Scientific* •Cardinal Health* •Cook* •Medtronic*	None	None	None	None	•Defendant, coronary interventions, 2019

© 2021 by the American College of Cardiology Foundation, the American Heart Association, Inc., and Society for Vascular Medicine.

Name	Employment	Representation	Consultant	Speakers Bureau	Ownership/ Partnership/ Principal	Personal Research	Institutional/ Organizational or Other Financial Benefit	Expert Witness
John Mulrow	Cardiology Clinic Cardiology Clinic of San Antonio—Cardiologist	Content Reviewer, ACC Geriatric Section Leadership Council	None	None	None	None	None	None
Aarti Patel	University of South Florida Morsani College of Medicine & James A. Haley VA Medical Center—Assistant Professor of Medicine Division of Cardiovascular Sciences; Tampa General Hospital—Director of Noninvasive Cardiology	Content Review, ACC Academic Section Leadership Council	None	None	None	None	None	None
Donna Polk	Harvard Medical School—Director, Cardiovascular Fellowship Program, Associate Professor of Medicine	Content Reviewer, ACC Program Directors and Graduate Medical Educators Section Leadership Council	None	None	None	None	•ACGME† •ASNC†	None
Harish Ramakrishna	Mayo Clinic College of Medicine—Professor of Anesthesiology; Mayo Clinic—Consultant, Division of Cardiovascular Anesthesia, Department of Anesthesiology and Perioperative Medicine	Content Reviewer, ACC Surgeons Section Leadership Council	None	None	None	None	None	None

© 2021 by the American College of Cardiology Foundation, the American Heart Association, Inc., and Society for Vascular Medicine.

Name	Employment	Representation	Consultant	Speakers Bureau	Ownership/ Partnership/ Principal	Personal Research	Institutional/ Organizational or Other Financial Benefit	Expert Witness
Nosheen Reza	University of Pennsylvania Health System—Fellow, Advanced Heart Failure and Transplant Cardiology	Content Reviewer, ACC Fellows in Training Section Leadership Council	None	None	None	None	None	None
Poonam Velagapudi	University of Nebraska Medical Center—Assistant Professor of Internal Medicine, Associate Program Director Cardiovascular Medicine Fellowship, Director Digital Innovation and Social Media Strategy	Content Reviewer, ACC Early Career Section Leadership Council	None	None	None	None	None	None
Michael N. Young	Dartmouth-Hitchcock Medical Center—Interventional Cardiology; Geisel School of Medicine at Dartmouth—Assistant Professor of Medicine	Content Reviewer, ACC Interventional Section Leadership Council	None	None	None	None	None	None

© 2021 by the American College of Cardiology Foundation, the American Heart Association, Inc., and Society for Vascular Medicine.

Name	Employment	Representation	Consultant	Speakers Bureau	Ownership/ Partnership/ Principal	Personal Research	Institutional/ Organizational or Other Financial Benefit	Expert Witness
Vlad G. Zaha	Harold C. Simmons Comprehensive Cancer Center, University of Texas Southwestern Medical Center—Medical Director of the Cardio-Oncology Program, Assistant Professor of Internal Medicine and Biomedical Engineering Division of Cardiology Advanced Imaging Research Center	Content Reviewer, ACC Cardio-oncology Section Leadership Council	None	None	None	•Cancer Prevention Research Institute of Texas* •NIH*	None	None

This table represents all healthcare relationships with industry and other entities by peer reviewers, including those not deemed to be relevant, at the time this document was under development. The table does not necessarily reflect relationships with industry at the time of publication. A person is deemed to have a significant interest in a business if the interest represents ownership of ≥5% of the voting stock or share of the business entity, or ownership of ≥$5,000 of the fair market value of the business entity; or if funds received by the person from the business entity exceed 5% of the person's gross income for the previous year. Relationships that exist with no financial benefit are also included for the purpose of transparency. Relationships in this table are modest unless otherwise noted. Please refer to http://www.acc.org/guidelines/about-guidelines-and-clinical-documents/relationships-with-industry-policy for definitions of disclosure categories or additional information about the ACCF Disclosure Policy for Writing Committees.

*Significant relationship.
†No financial relationship.

ABC indicates Association of Black Cardiologists; ACC, American College of Cardiology; ACGME, Accreditation Council for Graduate Medical Education; ACP, American College of Physicians; AHA, American Heart Association; ASNC, American Society of Nuclear Cardiology; BMS, Bristol-Myers Squibb; COCATS, Core Cardiovascular Training Statement; CSI, Cardiovascular Systems, Inc; CV, Cardiovascular; DSMB, data safety monitoring board; NIH, National Institutes of Health; SCAI, Society for Cardiovascular Angiography and Interventions; SVM; Society for Vascular Medicine; SVN, Society for Vascular Nursing; SVS, Society for Vascular Surgery; and VA, Veterans Affairs.

© 2021 by the American College of Cardiology Foundation, the American Heart Association, Inc., and Society for Vascular Medicine.
